# FUS::DDIT3 Fusion Protein in the Development of Myxoid Liposarcoma and Possible Implications for Therapy

**DOI:** 10.3390/biom14101297

**Published:** 2024-10-14

**Authors:** Xutong Hou, Wenjin Shi, Wenxin Luo, Yuwen Luo, Xuelin Huang, Jing Li, Ning Ji, Qianming Chen

**Affiliations:** State Key Laboratory of Oral Diseases, National Clinical Research Center for Oral Diseases, Research Unit of Oral Carcinogenesis and Management, Chinese Academy of Medical Sciences, West China Hospital of Stomatology, Sichuan University, Chengdu 610041, China; houxutong@stu.scu.edu.cn (X.H.); 2022224035178@stu.scu.esu.cn (W.S.); luowenxin@stu.scu.edu.cn (W.L.); yuwen_luo@scu.edu.cn (Y.L.); hxl_8121@scu.edu.cn (X.H.); lijing1984@scu.edu.cn (J.L.); qmchen@scu.edu.cn (Q.C.)

**Keywords:** chromosomal translocation, FUS::DDIT3 fusion protein, myxoid liposarcoma

## Abstract

The FUS::DDIT3 fusion protein, formed by the chromosomal translocation t (12;16) (q13;p11), is found in over 90% of myxoid liposarcoma (MLS) cases and is a crucial protein in its development. Many studies have explored the role of FUS::DDIT3 in MLS, and the prevailing view is that FUS::DDIT3 inhibits adipocyte differentiation and promotes MLS growth and invasive migration by functioning as an aberrant transcription factor that affects gene expression and regulates its downstream molecules. As fusion proteins are gradually showing their potential as targets for precision cancer therapy, FUS::DDIT3 has also been investigated as a therapeutic target. Drugs that target FUS::DDIT3 and its downstream molecules for treating MLS are widely utilized in both clinical practice and experimental studies, and some of them have demonstrated promising results. This article reviews the findings of relevant research, providing an overview of the oncogenic mechanisms of the FUS::DDIT3 fusion protein in MLS, as well as recent advancements in its therapy.

## 1. Introduction

**Figure 1:**
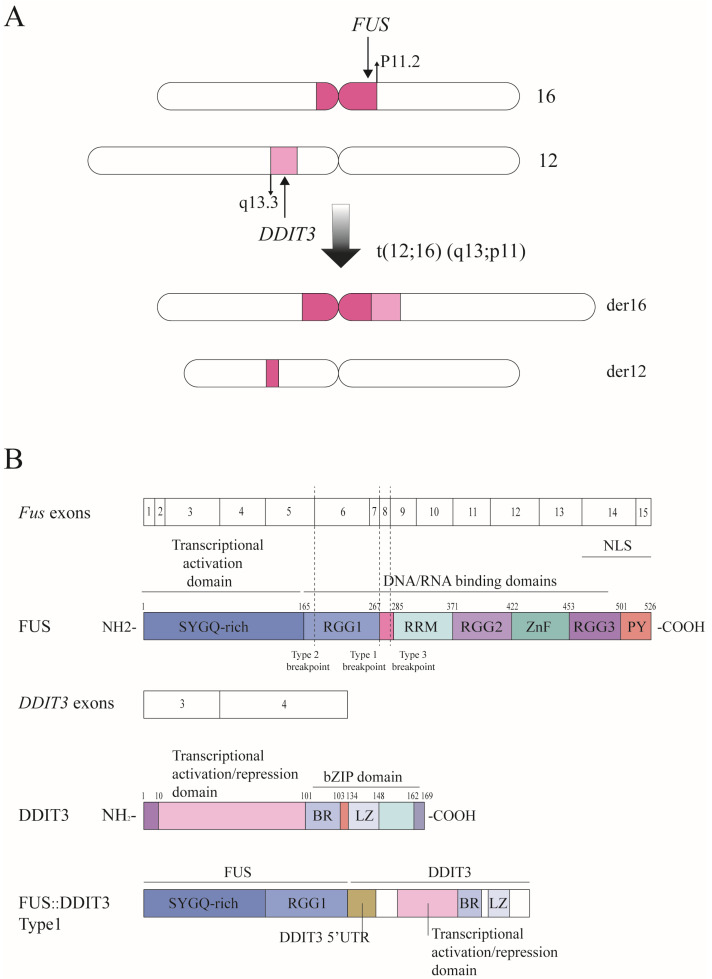
The FUS::DDIT3 fusion protein. (**A**) The chromosomal translocation t (12;16) (q13;p11) that cause the *FUS::DDIT3* fusion gene; (**B**) structure and domains of FUS, DDIT3, and FUS::DDIT3 (Type1). The RNA-binding domain of the FUS protein is replaced by DDIT3 in MLS. The FUS::DDIT3 fusion variant type1 retains the SYGQ-rich and RGG1 domains of FUS and also includes the in-frame amino acid sequence of a portion of the previously untranslated region (UTR) from DDIT3 exon2. Abbreviations: SYGQ-rich: serine–tyrosine–glycine–glutamine-rich domain; RGG: arginine–glycine–glycine box; RRM: RNA recognition motif; Znf: zinc finger; PY: proline–tyrosine nuclear localization signal; NLS: nuclear localization signal; bZIP domain: basic leucine zipper; BR: basic region; LZ: leucine zipper.

Myxoid liposarcoma (MLS) is the second most prevalent subtype of liposarcoma, constituting approximately 30–35% of all liposarcomas and around 10% of all sarcomas [1]. A population-based study shows that the overall survival rates for primary localized MLS at 5 and 10 years are 78% and 66%, respectively [2]. Though the standard treatment for MLS is surgery, there is still a tendency for local recurrence and distant metastasis after surgical resection, with incidence rates of 13–33% and 11–38%, respectively [3]. High-grade MLS, formerly known as round-cell liposarcoma, has a worse prognosis. It is a multicellular variant of myxoid liposarcoma, which contains more than 5% round cells. MLS is a translocation-related sarcoma, and over 90% of MLS cases are characterized by the chromosomal translocation t (12;16) (q13;p11), resulting in the fusion of the *FUS* (Fused In Sarcoma) and *DDIT3* (DNA damage-inducible transcript 3) genes at 12q13 and 16p11 to form the fusion gene *FUS::DDIT3* and to produce the FUS::DDIT3 fusion protein [4,5] (Figure 1A), although the relationship between FUS::DDIT3 and the round-cell components in MLS has not been found. A small part of MLS cases contain the EWSR1::DDIT3 fusion gene and fusion protein. However, due to the lack of separate studies on its pathogenic mechanism, this review only describes the role of FUS::DDIT3 in the development of MLS.

Chromosomal abnormalities strongly relate to tumor occurrence and development, with chromosomal translocation to form fusion genes being a common modality, driving 16.5% of cancer cases [6,7]. Among these, fusion genes of the *FET* (*FUS*, *EWSR1*, *TAF15*) family are an important part. *FET* genes as 5′partners and genes coding for transcription factors as 3′partners are fused to form fusion genes, which are translated into corresponding proteins, which are characteristic of many types of sarcomas and leukemias [8]. The most frequent FET family fusion oncoproteins are closely associated with the development of MLS and ewing sarcomas (ES) [9]. FET family fusion proteins are relatively diverse and have been studied for a long time. As the most numerous FET family fusion protein, the characteristic fusion protein of MLS, FUS::DDIT3, is increasingly being studied, and drugs related to it are being developed.

Fusion proteins can be used as cancer-specific diagnostic markers and potential drug and immunotherapy targets, and the study of fusion protein oncogenesis and related drug development is beneficial to the precision-targeted therapy of cancer [10,11]. The tyrosine kinase inhibitors (TKIs) imatinib and bosutinib target BCR-ABL1 in chronic myeloid leukemia (CML) significantly improve patient survival [12]; the tyrosine kinase inhibitor crizotinib demonstrates significant efficacy in treating *ROS1*-rearranged advanced non-small cell lung cancer [13]; and the tropomyosin receptor kinase (TRK) inhibitor larotrectinib exhibits significant and long-lasting antitumor activity in patients diagnosed with *TRK* fusion-positive tumors [14]. This review focuses on the oncogenic mechanism of FUS::DDIT3, the characteristic fusion protein of MLS, and the advancements in associated drug research.

## 2. Basic Structure and Main Functions of FUS::DDIT3

The FUS::DDIT3 protein, one type of FET family oncoproteins, is distributed in the nucleus of MLS cells and is considered an aberrant transcription factor [15]. The common feature of the FET family proteins is the N-terminal low complexity sequence (LCD), which is a prion-like motif, rich in aromatic and polar amino acids (Q/N/Y/S/G) and known as prion-like structure (PLD) [16]. The PLD is fused to the DNA-binding domain (DBD) of certain transcription regulators, resulting in the formation of the FET family oncoproteins [17]. FUS::DDIT3 is composed of the N-terminal domain of FUS and the full-length DDIT3, resulting in the RNA-binding domain of FUS being replaced by the basic leucine zipper (bZIP) domain of DDIT3 [18]. The N-terminal SYGQ-rich structural domain (LCD) of FUS acts as a transcriptional activation structural domain required for the oncogenic potential of the fusion protein [19] (Figure 1B).

FUS proteins are encoded by the *FET* gene family member *FUS* gene (also known as *TLS* gene) and are involved in the regulation of transcription and RNA processing [20]. FUS is a multi-domain protein with an N-terminal transcriptional activation domain, three arginine–glycine–glycine (RGG) boxes, an RNA recognition motif (RRM), and a zinc finger (ZnF), and the RGG2-ZnF-RGG3 domain is likely to be the major RNA-binding domain. At the C-terminus of FUS, there exists a non-classical nuclear localization signal (NLS) consisting of a proline–tyrosine NLS (PY-NLS) and the RGG3 structural domain [21] (Figure 1B). DDIT3 is a member of the CCATT enhancer-binding protein (C/EBP) transcription factor (TF) family. It is also known by several other names, including C/EBPζ, CHOP (C/EBP homologous protein), and GADD153 (growth arrest and DNA damage-inducible protein 153) [22]. Under normal physiological conditions, DDIT3 expression is notably low. However, it can be rapidly upregulated in response to various stressors such as endoplasmic reticulum stress, nutrient deprivation, DNA damage, cell growth arrest, and hypoxia [23]. The N-terminus of DDIT3 has a transcriptional activation/repression domain, which contains the basic region that mediates sequence-specific DNA binding (Figure 1B). Like other C/EBP proteins, it contains a conserved C-terminal domain, known as basic leucine zipper (bZIP), which is capable of forming heterodimers with other members of C/EBP and impairing its DNA binding activity, thereby acting as a negative regulator of other C/EBP proteins activity [24,25]. The C/EBP family exerts a controlling influence on the terminal differentiation of adipocytes and participates in the differentiation of other tissues [26]. DDIT3 binds to C/EBPβ to form an inactive heterodimer that blocks the binding activity of C/EBPβ to DNA, leading to loss of the adipocyte phenotype [27].

There are at least 12 variant types of *FUS* fusions with *DDIT3* identified to date [28]. The most prevalent among these is *FUS::DDIT3* type 2 (*FUS* exon 5 fuses to *DDIT3* exon 2), followed by type 1 (*FUS* exon 7 fuses to *DDIT3* exon 2) and type 3 (*FUS* exon 8 fuses to *DDIT3* exon 2) [29] (Figure 1B). However, these variants in the *FUS::DDIT3* structure did not significantly affect the level of adipogenesis and clinical outcomes such as histologic grades and survival rate [30].

FUS::DDIT3 has a significant association with the formation of human MLS, and a series of in vitro and in vivo studies have demonstrated that FUS::DDIT3 is a driver of MLS. Pérez-Losada J et al. [31] transfected the *FUS::DDIT3* gene into the mouse genome and observed an elevated expression of FUS::DDIT3 and most of the symptoms of MLS, confirming that FUS::DDIT3 overexpression is a determinant of MLS in humans. This is also the first in vivo evidence of a connection between fusion genes generated by chromosomal translocations and MLS in humans. Furthermore, Nicolò Riggi et al. [32] found that expression of the FUS::DDIT3 fusion protein in primary mesenchymal progenitor cells could establish MLS models. In another study, Rodriguez R et al. [33] expressed FUS::DDIT3 fusion protein in immortalized/transformed human mesenchymal stem cells, driving the formation of MLS. It has been observed in various studies that the FUS::DDIT3 fusion protein can prevent the differentiation of adipocytes and promote the growth, invasion, and migration of MLS [34,35].

## 3. Role and Mechanism of FUS::DDIT3 in MLS

### 3.1. Subsection FUS::DDIT3 and Adipocyte Differentiation

Adipogenesis consists of two phases: commitment and differentiation. The commitment is the mesenchymal stem cells (MSCs) differentiation into preadipocytes, which are morphologically similar to MSCs but lose the ability to differentiate into other cells. The differentiation is the stage in which preadipocytes differentiate into mature adipocytes. The differentiation of preadipocytes into mature adipocytes involves three stages: growth arrest, mitotic clonal expansion (MCE), and terminal differentiation [36].

Histologically, MLS is characterized by the prominent myxoid stroma and branching vessels. There is a proliferation of small, round-to-oval-shaped, non-adipocytic mesenchymal cells mixed with variable numbers of immature lipoblasts of different stages set in stroma [1]. Lipoblasts are conceptually the precursor or immature form of adipocytes and are histologically defined as mononuclear or multinucleated cells containing lipid droplets [37]. FUS::DDIT3 inhibits the process of adipogenic differentiation, which explains the primitive adipocytic morphology in MLS.

**Figure 2:**
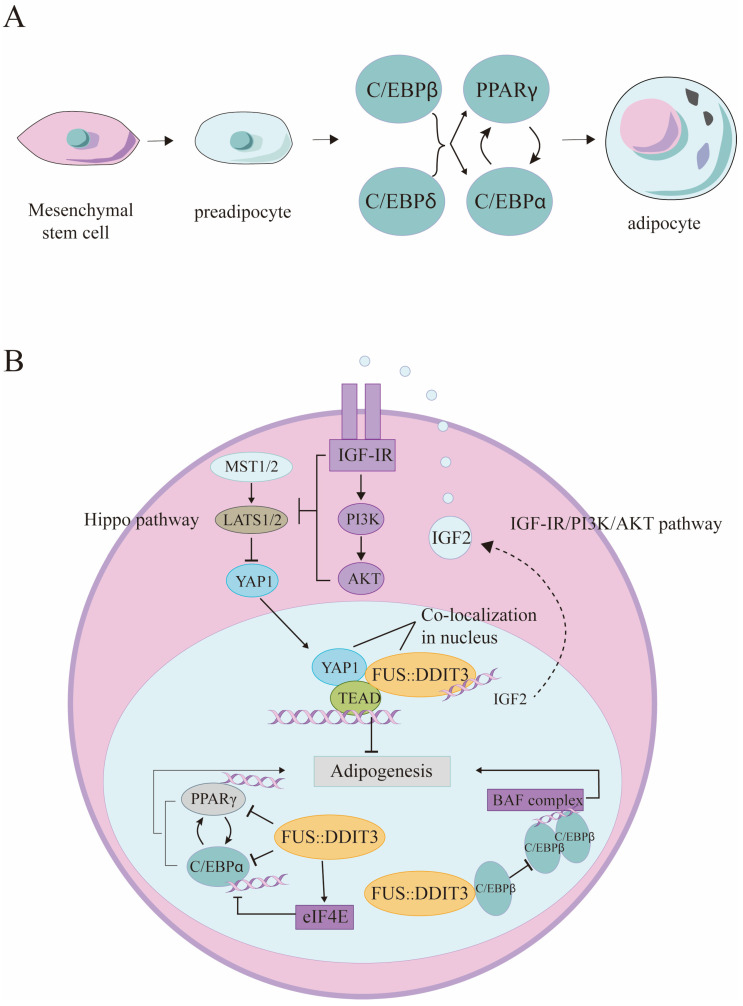
FUS::DDIT3 inhibits adipogenesis. (**A**) Normal mesenchymal stem cells differentiation program and relative regulation of adipogenic factors. C/EBPβ and C/EBPδ regulate the expression of PPARγ and C/EBPα. PPARγ activates the promoter of the gene encoding C/EBPα and vice versa, creating a positive feedback loop. (**B**) FUS::DDIT3 blocks adipogenesis via the three main signal pathways: (i) inhibiting PPARγ and C/EBPα translation; (ii) preventing the combination of C/EBPβ homodimers and BAF complex; (iii) FUS::DDIT3 co-localizes and physically binds to YAP1 and TEAD in the nucleus, and together they drive IGF2 expression, leading to an IGF-II/IGF-IR transactivation loop.

Differentiation of preadipocytes to adipocytes is regulated by a network of transcription factors that centers on two major adipogenic factors, PPAR γ and C/EBPα, which regulate each other’s expression and are involved in a single pathway of adipogenesis, with PPAR γ being the dominant factor [38]. FUS::DDIT3 prevents the differentiation of preadipocytes to adipocytes by repressing the active sequences of the PPAR γ and C/EBPα promoters and thereby inhibiting their expression, resulting in the accumulation of immature adipocytes of different stages. In addition, FUS::DDIT3 induces the expression of eukaryotic translation initiation factor eIF4E, which converts C/EBPα to the truncated p30-C/EBPα isoform, negatively affecting adipocyte differentiation and contributes to the attenuation of the positive feedback loop between C/EBPα and PPAR γ, further attenuating the normal adipocyte differentiation program [35] (Figure 2B). During adipose differentiation, C/EBPβ and C/EBP δ of the C/EBP family are expressed earlier than PPAR γ and C/EBPα and regulate the expression of both. The basic leucine zipper domain of FUS::DDIT3 heterodimerizes with C/EBPβ, directly preventing the binding and transactivation of C/EBPβ to its target genes and inhibiting C/EBPβ-mediated adipogenesis [39] (Figure 2A).

BAF (BRG1/BRM-associated factor) complex, or mSWI/SNF complex, an ATP-dependent chromatin remodeling complex, is also an important target for FUS::DDIT3-induced MLS in addition to PPAR γ and C/EBPα. ATP-dependent chromatin remodeling is an important mechanism of DNA compaction and decompaction within the nucleus, ensuring that DNA in chromatin has functions involved in replication, selective gene expression, DNA damage repair, and recombination [40]. The BAF complex also regulates normal gene expression by an antagonistic interaction with polycomb repressive complex 2 (PRC2) [41]. The normal BAF complex interacts with the adipogenic factors PPAR γ and C/EBP and is involved in gene activation at the early and late stages of adipocyte differentiation, which is necessary for normal adipocyte differentiation. The BAF complex engages in an interaction with the transactivation element III (TE-III) of C/EBPα and is involved in C/EBPα-mediated adipogenesis. Additionally, BAF chromatin remodeling enzymes are indispensable for the activation of the adipogenic gene program by C/EBPα or C/EBPβ [42,43]. During adipogenesis, BAF may activate PPAR γ regulators and lipogenic marker genes that are subsequently expressed during differentiation by promoting the function of the preinitiation complex (PIC) [43]. Recent studies support the idea that FUS::DDIT3 drives competition between the generation of C/EBPβ homodimers and FUS::DDIT3-C/EBPβ heterodimers in MLS. FUS::DDIT3 expression attenuates the formation of C/EBPβ homodimers that bind to enhancers and target BAF complexes, which inhibits C/EBPβ chromatin binding and C/EBPβ-mediated recruitment of the BAF complex, leading to reduced chromatin activation and decreased expression of adipose genes (genes central to lipid, cholesterol, and steroid biosynthesis, such as IRS1, LPIN1, and STAT5B) and upregulated expression genes associated with cell cycle and growth pathway (e.g., CXCL8, TRIB3, and PTX3) [44] (Figure 2B).

In addition to the above two pathways, FUS::DDIT3 also blocks adipogenesis by regulating the Hippo pathway [45]. The Hippo pathway is a highly conserved kinase cascade that regulates organ size and cell differentiation, and the transcriptional coactivator Yes-associated protein 1 (YAP1) is a transcription regulator and effector molecule of this pathway involved in tissue growth and tumorigenesis [46]. When serine/threonine kinase MST1/2 and large tumor suppressor 1/2 (LATS1/2) kinases are active, the Hippo pathway is turned on, which leads to cytoplasmic retention and proteasomal degradation of YAP1; conversely, when Hippo signaling is “off”, YAP1 translocates to the nucleus and binds to transcription factors (e.g., TEAD), thereby regulating the expression of target genes [47]. Research has demonstrated a strong correlation between the enhanced activity of YAP1 and the development of MLS, and inhibition of its activity can effectively inhibit the growth of MLS, which is realized through FUS::DDIT3 [46]. However, recurrent genetic alterations affecting components of the Hippo pathway have not been identified in MLS [48]. Further signaling pathway studies have revealed that FUS::DDIT3 drives IGF2 expression, leading to an IGF-II/IGF-IR transactivation loop [49]. FUS::DDIT3-mediated IGF-IR/PI3K/AKT signaling leads to the closure of the Hippo pathway, and downstream unphosphorylated YAP1 translocates to the nucleus. FUS::DDIT3 co-localizes and physically binds to YAP1 and TEAD in the nucleus, and together, they regulate the expression of oncogenes associated with proliferation, cell cycle progression, apoptosis, and adipogenesis, and prevent terminal adipogenic differentiation [50] (Figure 2B). In addition, YAP1 regulates the proliferation and differentiation of preadipocytes by altering the expression of PPAR γ. It was found that overexpression of YAP1 inhibited the expression of PPAR γ, thereby inhibiting the differentiation of preadipocytes [51].

### 3.2. FUS::DDIT3 and the Growth of MLS

Cyclin-dependent kinases (CDKs) control the transition between different cell cycles and participate in the proliferation and growth of tumor cells through cell cycle regulation, playing a key role in tumor pathogenesis [52]. Cell cycle proteins are categorized according to their expression and involvement in cell cycle control: cyclin E, which is involved in G1/S control; cyclin A (S-phase) and cyclin B (M-phase); cyclin D, which controls the entry of the cell cycle into the G1 phase [53]. FUS::DDIT3 regulates the growth of MLS by affecting cell cycle progression. Normally, *DDIT3* is transcribed at low levels but is elevated under cellular stress conditions and is involved in the stress response and cancer process by inducing cell cycle arrest and apoptosis [25]. In MLS cell lines, FUS::DDIT3 has a role opposite to that of DDIT3. DDIT3 acts at the G1/S checkpoint to cause cell growth arrest, whereas its fusion protein, FUS::DDIT3, not only fails to cause growth arrest but also hinders the action of DDIT3 and thus plays a role in MLS development [54].

**Figure 3:**
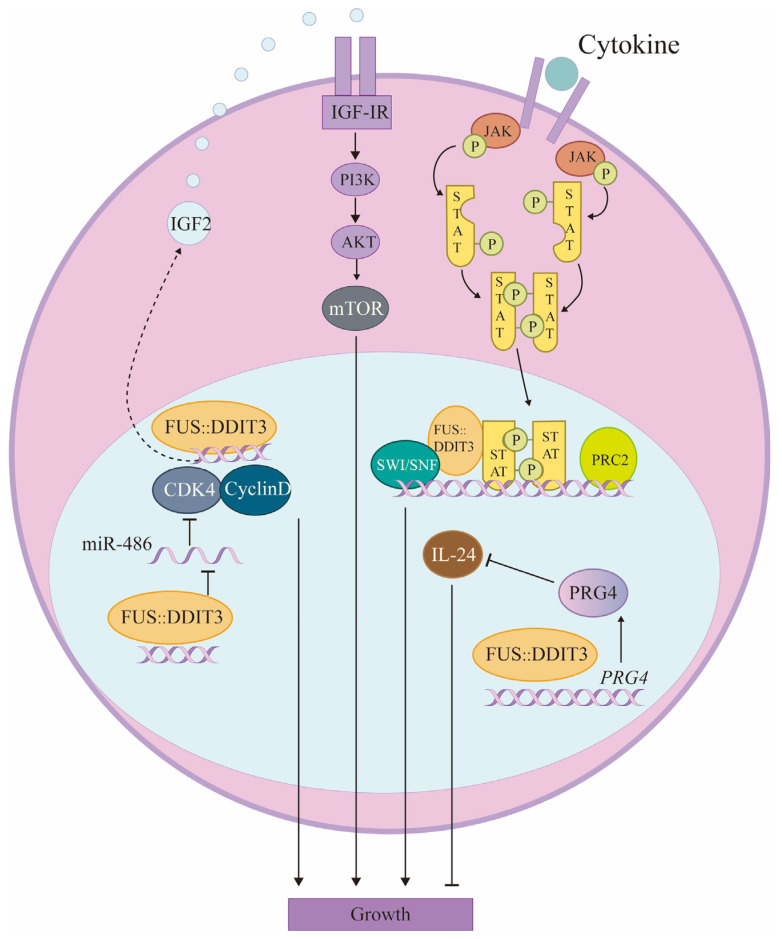
FUS::DDIT3 promotes MLS growth. FUS::DDIT3 is involved in IGF-IR/PI3K/AKT and JAK/STAT signaling pathways, miR-486/CDK4 axis, and IL-24 expression to promote MLS growth.

In MLS cell lines, strong overexpression of cyclin D1 and E related to the G1 cell cycle and their associated kinases CDK2 and CDK4 have been detected, whereas cyclin A specific for the S and G2 phases of the cell cycle is lowly expressed in these tumors. Cell cycle dysregulation may constitute a significant factor in the pathogenesis of MLS, and evidence suggests that FUS::DDIT3 is implicated in this dysregulation [55]. FUS::DDIT3 binds to cell cyclin-dependent kinase 2 (CDK2) via the DDIT3 portion. CDK2 translocates to nuclear structures defined by FUS::DDIT3, and the interaction between the two alters the binding affinity of CDK2, which can lead to altered phosphorylation patterns and regulation of cytoskeletal or other proteins [56]. In addition, CDK4 was found to be overexpressed in MLS [57]. The *CDK4* gene, located on chromosome 12q13, encodes a protein that acts as an intermediary between extracellular signaling pathways and the cell cycle. When CDK4 binds to cyclin D, it promotes DNA synthesis and cell proliferation [58]. FUS::DDIT3 inhibits miR-486 expression, thereby promoting CDK4 expression and regulating MLS cell proliferation and apoptosis via the miR-486/CDK4 axis [59] (Figure 3). The impact of FUS::DDIT3 on cell cycle regulation requires further investigation to analyze the effects of FUS::DDIT3 on individual cell cycle regulators.

Interleukin-24 (IL-24) is a cytokine with potential antitumor effects that can affect a wide range of cancers. IL-24 selectively inhibits tumor cell growth, invasion, metastasis, and angiogenesis, induces cancer-selective apoptosis, stimulates the anticancer immune response, sensitizes cancer cells to treatment, and exerts antitumor effects through multiple pathways [60]. In MLS, IL-24 expression is decreased, and knockdown of FUS::DDIT3 results in increased IL-24 expression and inhibition of tumor cell growth [61]. Mechanically, the proteoglycan 4 (PRG4), also known as the downstream of the liposarcoma-associated fusion oncoprotein 54 (DOL54), is one of the downstream molecules of FUS::DDIT3 [62]. And in MLS, the presence of PRG4 has been observed to sustain the proliferation of tumor cells, which is achieved through the suppression of IL-24, a cytokine known for its antitumor properties [63] (Figure 3).

In addition, as mentioned previously, the IGF-IR/PI3K/Akt pathway is activated in MLS. The activation mechanisms include increased IGF-1R and activation of the PIK3CA mutation [64]. MLS cell proliferation and viability are significantly dependent on PI3K-mediated signaling, both in vitro and in vivo [65]. In contrast, overexpression of FUS::DDIT3 induces the activation of aberrant IGF-IR/PI3K/Akt signaling activity. mTOR, a downstream target of Akt, exhibits increased levels of phosphorylation in MLS [49]. The mTOR signaling pathway, frequently activated in tumors, has a significant impact on tumor metabolism, cell proliferation, and immune cell differentiation [66]. Aberrant activation of the IGF-IR/PI3K/Akt pathway seems to contribute to the activation of mTOR, which results in the growth and proliferation of MLS tumor cells (Figure 3).

In addition to the three ways mentioned above, cancer stem cells (CSC) are a small subpopulation of cells within a tumor, possessing the capabilities of self-renewal, differentiation, and tumor formation. CSCs are involved in tumor development, cell proliferation, and metastatic dissemination and demonstrate resistance to chemotherapy and radiotherapy [67]. MLS contains CSC-like cells that form non-adherent spheroids, exocytose Hoechst dye, and resist chemotherapeutic agents; the JAK-STAT pathway is active in MLS and regulates the size of CSC-like subpopulations [68]. Subsequent studies have confirmed that FUS::DDIT3 expression leads to aberrant activation of the JAK-STAT pathway through its interaction with phosphorylated STAT3 (pSTAT3) and that SWI/SNF and PRC2 complexes may be implicated in this pathway. The clinical significance of JAK-STAT in MLS treatment remains to be determined by further studies [69] (Figure 3).

### 3.3. FUS::DDIT3 and Metastasis of MLS

**Figure 4:**
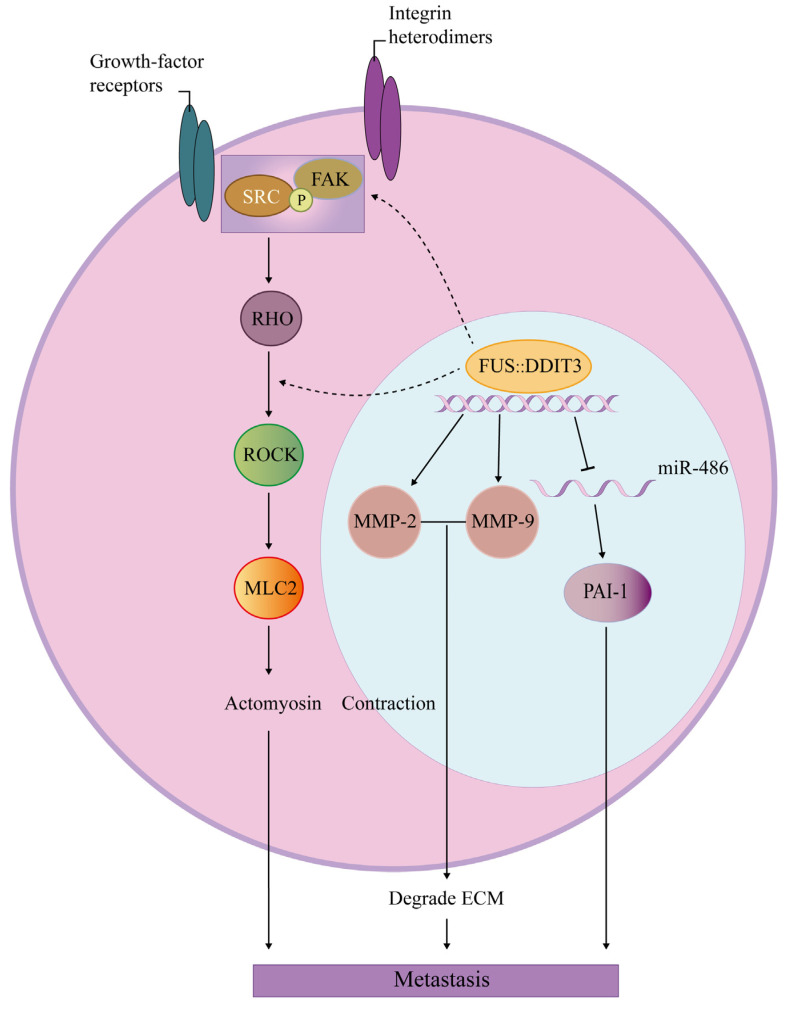
FUS::DDIT3 promotes MLS metastasis. The mechanisms by which FUS::DDIT3 promotes MLS metastasis include increasing the expression of matrix metalloproteinases, activating the SRC/FAK/RHO/ROCK signaling axis, and inducing the expression of PAI-1 by inhibiting the expression of miR-486.

MLS, with a high incidence of bone metastases, which is associated with FUS::DDIT3 fusion, exhibits a distinct metastatic pattern in contrast to other soft tissue sarcomas [70]. Matrix metalloproteinases (MMPs) are a class of enzymes that play a role in tissue remodeling and repair, degradation of extracellular matrix (ECM) components, and promotion of tumor cell invasion and proliferation [71]. FUS::DDIT3 transactivates the *MMP-2* and *MMP-9* promoters, and this activation is mainly mediated by AP-1, NF-κB, and C/EBP-β sites of two matrix metalloproteinases [72]. FUS::DDIT3 induces MLS metastasis through enhanced transcriptional activation of two matrix metalloproteinases, and MMP-2, in particular, is crucial for FUS::DDIT3-mediated cell migration and invasion (Figure 4).

SRC and focal adhesion kinase (FAK) are non-receptor tyrosine kinases. The SRC-FAK pathway is activated in a variety of tumors and generates signals that lead to tumor growth and metastasis [73]. Subject to various stimuli, including integrins, FAK undergoes autophosphorylates at specific tyrosine (Y) residue Y397, leading to the formation of a binding site with a strong affinity for SRC, which in turn triggers autophosphorylation of SRC at Y419. Once fully activated, SRC can enhance the activation of FAK through phosphorylation of its C-terminal and catalytic domains [74]. Rho GTPases, functioning as molecular switches, alternate between an active state when bound to GTP and an inactive state when bound to GDP, regulating actomyosin polymerization and organization into distinct cytoskeletal structures, which is crucial for cell migration [75]. It is found that FUS::DDIT3 increases the phosphorylation level of SRC and FAK and the overall protein level of FAK. SRC/FAK is an upstream mediator of RHO/ROCK signaling activation, and RHO further activates RHO-associated coiled-coil-containing protein kinase (ROCK), leading to elevated levels of phosphorylation of myosin light chain 2 (MLC2), which promotes the contractile capacity of actomyosin and thus facilitates MLS invasion [76]. Mechanistic studies on the activation of the SRC/FAK/RHO/ROCK signaling axis by FUS::DDIT3 to promote MLS metastasis provide a theoretical basis for its therapeutic strategy (Figure 4).

Plasminogen activator inhibitor-1 (*PAI-1*) is a target gene of miR-486. It controls protein hydrolysis activity and cell migration during angiogenesis, and its high level of expression promotes tumor invasion and angiogenesis [77]. MiRNAs regulate biological processes by negatively regulating the expression of target genes at the mRNA level [78]. In MLS, FUS::DDIT3 can induce PAI-1 expression by inhibiting miR-486 expression, thus promoting tumor metastasis [79] (Figure 4).

## 4. DDIT3 Detecting in MLS

The abnormal expression of DDIT3 may induce adipocyte differentiation and block the late stage of adipogenesis. Most well-differentiated/dedifferentiated liposarcoma (WDLS/DDLS) cases contain amplified fragments of chromosome 12q13-15, which contains DDIT3. MLS carries a rearranged *DDIT3* fused with *FUS* or *EWSR1* [80]. Mantilla JG et al. [81] have observed *DDIT3* amplification in 33% of dedifferentiated liposarcoma cases and was significantly associated with the presence of myxoid liposarcoma-like features compared with cases without amplification. The forced expression of FUS::DDIT3 and normal DDIT3 induced the transformation of HT1080 cells into MLS-like morphology, including the morphology of capillary network similar to MLS and gene expression pattern [82].

Fluorescence in situ hybridization (FISH) detection of *DDIT3* gene rearrangement has been used in clinical practice and has become the gold standard for molecular diagnosis of MLS [83]. However, the FISH detection method is expensive. Reverse transcription polymerase chain reaction (RT-PCR) is commonly used to detect gene fusion. However, there are at least 14 different FUS::DDIT3 and EWSR1::DDIT3 variants, making it difficult to diagnose MLS [84]. Some studies have reported that DDIT3 immunohistochemistry (IHC) has high specificity and sensitivity in MLS.

FUS::DDIT3 has the peptide sequence corresponding to the normally untranslated *DDIT3* exon 2 and parts of exon 3 (5′-UTR). Oikawa K et al. [85] have generated monoclonal antibodies against this unique peptide sequence, which can react with FUS::DDIT3 fusion proteins but not react with normal FUS and DDIT3 proteins by Western blot analysis. DDIT3 IHC can recognize almost all variants of FUS::DDIT3 (except type 4). In most MLS cases, diffuse nuclear staining of tumor cells can be observed, and non-tumor cells, such as endothelial cells, are not identified [86]. It recognizes the N-terminus of DDIT3, so regardless of the fusion partner (*FUS* or *EWSR1*), the antibody is expected to recognize tumor-specific oncoproteins. Compared with FISH and RT-PCR techniques used to identify DDIT3 rearrangements, IHC may provide a faster and cheaper diagnostic confirmation method [87]. High-grade MLS (round-cell liposarcoma) with pure round-cell morphology may be difficult to distinguish from other round-cell sarcomas. DDIT3 IHC can distinguish high-grade MLS from other round-cell sarcomas with a sensitivity of 96% and a specificity of 98% [88]. MLS, even high-level round-cell cases, have uniform karyotypes. A few MLSs showed nuclear pleomorphism and would be confused with other sarcoma types. DDIT3 analysis will solve this problem because these sarcoma types do not have DDIT3 fusion [89]. However, the focal expression of DDIT3 occurs in 1.5–5% of non-MLS tumors and is difficult to define. The significance of focal staining needs to be considered according to the specific situation [86].

## 5. FUS::DDIT3-Mediated Targeted Drugs

The primary treatment for MLS is radical surgery and radiotherapy. Although MLS is more sensitive to chemotherapy than other types of liposarcoma, the treatment options remain limited for patients with recurrent and metastatic conditions [90]. MLS has few mutations aside from the *FUS::DDIT3* fusion, making targeting FUS::DDIT3 or its downstream regulators an effective therapeutic strategy [91] (Table 1).

### 5.1. Drugs under Clinical Experiments Targeting FUS::DDIT3 and Its Downstream Regulators

Clinically, anthracyclines are used for first-line treatment in advanced and metastatic MLS [92,93]. Trabectedin is a second-line therapeutic agent for MLS with favorable results in cases of advanced MLS cases that fail standard therapy [94]. Trabectedin also has a favorable risk/efficacy profile in combination with other drugs [95]. In advanced metastatic cases, the combination of trabectedin with low-dose radiation therapy has an excellent remission rate (NCT02275286) [96]. Some clinical trials have demonstrated that this combination could potentially serve as an alternative to anthracycline-based chemotherapy in MLS [97,98]. Trabectedin is a multifunctional oncostatic drug, and one of its main targets of action is FUS::DDIT3 [99]. It blocks the binding of FUS::DDIT3 to the DNA promoter, reduces the oncoproteins FUS::DDIT3 type I and II, activates the expression of the lipogenic factors c/EBP α and β, and stimulates the reaction of adipogenesis and the re-emergence of mature lipogenic cells [100].

However, some studies have identified trabectedin resistance. Uboldi S et al. [101] established the first trabectedin-resistant MLS cell line, 402-91/ET. In this resistant cell line, trabectedin failed to activate the transcription of adipogenesis-related genes (e.g., c/EBPα and β). In a mouse model of human MLS, one of the reasons for resistance was found to be the loss of 4p15.2, 4p16.3, and 17q21.3 chromatin as a result of prolonged treatment, and the downregulation of the expression of genes in these chromatin bands [102]. For example, the *UVSSA* (UV-stimulated scaffolding protein A) gene on 4p16.3 plays a role in a process called transcription-coupled nucleotide excision repair (TC-NER). The loss of *UVSSA* leads to defects in TC-NER, which is crucial for active genes such as those involved in trabectedin-induced adipogenesis. This leads to a deregulation of the pathway, leading to resistance of cancer cells to trabectedin [103].

### 5.2. Drugs under Preclinical Experiments Targeting FUS::DDIT3 and Its Downstream Regulators

PPARγ (peroxisome proliferator-activated receptor gamma) carries out the central role in adipogenesis and can be reduced by FUS::DDIT3. The use of ligands for PPAR γ induces terminal differentiation of liposarcoma cells and thus represents a new approach to liposarcoma treatment [104]. Pioglitazone, a member of the thiazolidinedione (TDZ) class of antidiabetic drugs, functions as an agonist ligand for PPAR γ. It binds to PPARγ and acts as a potent regulator of adipocyte development [105]. Pioglitazone reactivates adipocyte differentiation. In a mouse model, the combination of pioglitazone and trabectedin was employed to counteract resistance to trabectedin treatment for MLS [106]. The clinical trial of pioglitazone in combination with trabectedin for MLS, NCT04794127, is in phase II. In addition, a patient with advanced metastatic MLS did well with efatutazone, also a PPAR γ agonist (NCT00408434) [107]. A clinical trial (NCT02249949) is applying efatutazone hydrochloride (efatutazone dihydrochloride) to treat patients with previously treated MLS that is not amenable to surgical removal. What is more, combining trabectedin with PPARγ agonists shows the possibility of treatment. Rosiglitazone enhanced trabectedin-induced adipogenesis and survival in an MLS mouse model [108].

FUS::DDIT3 closes the Hippo pathway, and downstream, YAP1 translocates to the nucleus, regulating gene expression with FUS::DDIT3. YAP1 has been identified as a molecular target for therapeutic intervention in MLS, and pharmacological inhibition of YAP1 activity with verteporfin has been demonstrated to inhibit MLS cell viability both in vitro and in vivo [45]. Overexpression of FUS::DDIT3 leads to activation of IGF-IR/PI3K/Akt signaling. The IGF-IR ATP inhibitor, NVP-AEW541, and the IGF-IR non-ATP inhibitor, PPP, induce apoptosis and decrease mitogenic activity. This results in reduced cellular activity in MLS cell lines and a decrease in tumor volume in an in vivo model of chick CAM for MLS [49]. The PI3K inhibitor buparlisib (BKM120) reduces MLS cell viability in vitro by inducing apoptosis and has been confirmed in an animal model with a significant reduction in tumor volume [65]. Dolatabadi S et al. [68] have found that a subpopulation with cancer stem cell (CSC) characteristics existed in MLS and that JAK-STAT signaling was active in MLS cell lines and regulated CSC characteristics, leading to the development of drug resistance in the tumor cells; the use of ruxolitinib, an inhibitor of JAK-STAT, can reduce the number of CSC-characteristic chemoresistant cells. The combination of ruxolitinib with the chemotherapeutic agent adriamycin for the treatment of targeted proliferating cells and cells with CSC characteristics provides a new means of circumventing chemoresistance in the treatment of MLS patients [69]. The clinical study of JAT inhibitor itacitinib treating advanced or metastatic sarcoma (including MLS) is in the first stage (NCT03670069).

Willems SM et al. [109] have found that Src pathway activation-associated kinases are active in MLS. SRC inhibitor dasatinib reduces the viability of MLS cells and shows additive effects with cytotoxic drugs [110]. Inhibition of the SRC/FAK/RHO/ROCK signaling axis, achieved through the use of SRC inhibitor dasatinib and the FAK inhibitor PF-573228, reduces MLS invasiveness and prevents the invasion of CSC-rich subpopulations, thereby decreasing the self-renewal and invasive potential of MLS; furthermore, the ROCK inhibitor RKI-1447 has been shown to completely abrogate invasion in cells expressing FUS::DDIT3.

## 6. Conclusions and Perspectives

After three decades of research, it has been demonstrated that the FUS::DDIT3 fusion protein is a key protein in the development of MLS. A large number of experimental studies have shown that FUS::DDIT3 fusion proteins can affect tumor development by regulating the proliferation, differentiation, invasion, migration, and other biological functions of MLS cells, but the detailed regulatory mechanisms and closely related signaling pathways need to be further investigated. Currently, there are fewer drugs for MLS, especially metastatic MLS, and numerous MLS-related drugs are in the preclinical or experimental stage of research, which still need to be put into large-scale animal experiments and clinical trials. However, the study of the FUS::DDIT3 fusion protein and its related signaling pathway, as well as the study of targeted drugs against this fusion protein and the corresponding signaling pathway proteins, will be an important direction for the research of MLS and its treatment.

## Figures and Tables

**Table 1:** Drugs targeting FUS::DDIT3 and its downstream regulators.

Drugs	Target	Singling Pathway	Clinical or Preclinical Experiment
Trabectedin	FUS::DDIT3	/	Phase I/II (NCT02275286)
Pioglitazone	PPARγ	/	Phase II (NCT04794127)
Efatutazone	PPARγ	/	Phase I (NCT00408434) Phase II (NCT02249949)
Itacitinib	JAK	JAK-STAT	Phase I (NCT03670069)
Verteporfin	YAP1	Hippo	Vivo and vitro experiments
Picropodophyllin (PPP)	IGF-IR	IGF-IR/PI3K/AKT	Vivo and vitro experiments
Buparlisib (BKM120)	PI3K	IGF-IR/PI3K/AKT	Vivo and vitro experiments
Ruxolitinib	JAK	JAK-STAT	Vitro experiment
Dasatinib	SRC	SRC/FAK/RHO/ROCK	Vitro experiment
NVP-AEW541	IGF-IR	IGF-IR/PI3K/AKT	Vivo and vitro experiments
PF-573228	FAK	SRC/FAK/RHO/ROCK	Vitro experiment
RKI-1447	ROCK	SRC/FAK/RHO/ROCK	Vitro experiment

This table summarizes information on drugs under clinical and preclinical experiments targeting FUS::DDIT3 and its downstream regulators.
